# Combined Microbiome and Metabolome Analysis Reveals a Novel Interplay Between Intestinal Flora and Serum Metabolites in Lung Cancer

**DOI:** 10.3389/fcimb.2022.885093

**Published:** 2022-05-02

**Authors:** Sai Chen, Rong Gui, Xiong-hui Zhou, Jun-hua Zhang, Hai-ye Jiang, Hai-ting Liu, Yun-feng Fu

**Affiliations:** ^1^Department of Blood Transfusion, The Third Xiangya Hospital of Central South University, Changsha, China; ^2^Department of Laboratory Medicine, The Third Xiangya Hospital of Central South University, Changsha, China

**Keywords:** lung cancer, intestinal microorganism, intestinal flora, serum metabolites, biomarker

## Abstract

As the leading cause of cancer death, lung cancer seriously endangers human health and quality of life. Although many studies have reported the intestinal microbial composition of lung cancer, little is known about the interplay between intestinal microbiome and metabolites and how they affect the development of lung cancer. Herein, we combined 16S ribosomal RNA (rRNA) gene sequencing and liquid chromatography-mass spectrometry (LC-MS) technology to analyze intestinal microbiota composition and serum metabolism profile in a cohort of 30 lung cancer patients with different stages and 15 healthy individuals. Compared with healthy people, we found that the structure of intestinal microbiota in lung cancer patients had changed significantly (Adonis, *p* = 0.021). In order to determine how intestinal flora affects the occurrence and development of lung cancer, the Spearman rank correlation test was used to find the connection between differential microorganisms and differential metabolites. It was found that as thez disease progressed, L-valine decreased. Correspondingly, the abundance of *Lachnospiraceae_UCG-006*, the genus with the strongest association with L-valine, also decreased in lung cancer groups. Correlation analysis showed that the gut microbiome and serum metabolic profile had a strong synergy, and *Lachnospiraceae_UCG-006* was closely related to L-valine. In summary, this study described the characteristics of intestinal flora and serum metabolic profiles of lung cancer patients with different stages. It revealed that lung cancer may be the result of the mutual regulation of L-valine and *Lachnospiraceae_UCG-006* through the aminoacyl-tRNA biosynthesis pathway, and proposed that L-valine may be a potential marker for the diagnosis of lung cancer.

## Introduction

Lung cancer has the characteristics of high morbidity and high mortality, which seriously endangers human health and quality of life. As the leading cause of cancer death, there would be an estimated 2.2 million new cases and 1.79 million deaths worldwide in 2020 (Sung et al., 2021). Many early-stage lung cancer patients have no symptoms or only have mild symptoms with no specificity, making it difficult to attract attention. However, the diagnosis of lung cancer requires complicated procedures such as pathological biopsy and imaging examinations, and there is a lack of simple bedside detection methods. Therefore, many lung cancer patients are often in the middle or advanced stages when they are diagnosed, so there are always different degrees of cancer metastasis, leading to a poor prognosis (Rudin et al., 2021). The current non-invasive diagnostic method for lung cancer is mainly liquid biopsy. There is a small amount of circulating tumor cells (CTC) shed from the tumor site in the blood of cancer patients, and necrotic cancer cells release a small amount of circulating tumor DNA (ctDNA) into the blood; therefore, it can help to judge the occurrence of cancer by detecting CTC and ctDNA. In addition, non-invasive biomarkers based on protein and microRNA have also been widely studied (I and Cho, 2015; Farooq and Herman, 2020). In recent years, a growing number of studies have discovered the connection between intestinal flora and disease diagnosis, treatment, and prognosis. At present, many studies involve the sequencing of gut microbes in lung cancer patients, and Zheng et al. (2020) have developed an operational taxonomic unit (OTU)-based prediction model for the early diagnosis of lung cancer. However, relying on single omics for prediction does not seem to be sufficient, and so far, the connection and interaction between the gut microbiome and metabolome of lung cancer patients with different stages have not been recorded.

A cohort study found that the diversity of intestinal flora was at a similar level in healthy children and adults, but the composition and function of the microbiome were different (Wen et al., 2019). Herein, we assume that there are differences in the composition or diversity of the intestinal flora of lung cancer patients with different stages. We recruited 30 different-stage lung cancer patients and 15 healthy individuals and performed the corresponding detection and analysis on their stool and serum specimens. The composition of the intestinal flora and serum metabolites was compared by bioinformatics analysis. We are trying to combine microbiology and metabolomics to find out the pathogenesis of lung cancer and potential biomarkers, so as to provide new insights for the diagnosis and treatment of lung cancer in the future.

## Materials and Methods

### Participants and Sample Collection

Thirty newly diagnosed lung cancer patients from Hunan Cancer Hospital and 15 individuals undergoing physical examination from the Health Management Center of the Third Xiangya Hospital of Central South University were included in our study. Stool and serum samples were collected according to the protocol approved by the Ethics Committee of the Third Xiangya Hospital of Central South University, and written informed consent from all participants was obtained. The exclusion criteria were as follows: 1) individuals with primary carcinoma of other organs, 2) individuals suffering from other cancers, and 3) individuals receiving antibiotics or probiotics within the past 3 months. The control group was matched according to age and sex ratio.

Fresh stool samples of each participant were collected and then placed on ice immediately. The temperature was ensured to be below 4°C, and the samples collected were stored at −80°C within 1 h until DNA extraction. Intravenous blood collection was carried out by professional nurses in strict accordance with aseptic standard procedures. Serum was collected by centrifugation and stored at −80°C until being tested.

### Microbial DNA Extraction and Sequencing

QIAamp 96 Power Fecal QIAcube HT kit (Qiagen, Hilden, Germany) was used to extract total DNA from stool samples according to the manufacturer’s instructions. NanoDrop2000 (Thermo Fisher, USA) and 1% agarose gel electrophoresis were used to detect the concentration and quality of DNA. Barcode-specific primers (primers 5′-TACGGRAGGCAGCAG-3′ and 5′-AGGGTATCTAATCCT-3′) were used to perform PCR amplification on the 16S V3–V4 region.

The PCR products were separated on a 2% agarose gel and further purified using AxyPrep DNA Gel Extraction Kit (Axygen Biosciences, Union City, CA, USA). The purified PCR products were quantified using Qubit dsDNA Assay Kit (Life Technologies, Waltham, MA, USA). Finally, equal amounts of samples were mixed according to the concentration of PCR products, and NovaSeq PE250 paired-end sequencing was performed.

### 16S rRNA Sequencing Result Data Processing

The Trimmomatic software (Bolger et al., 2014) was used to remove impurity from the original double-ended sequence (FASTQ format), and the FLASH software (Reyon et al., 2012) was used to merge after impurity removal. Then, sequences containing ambiguous bases, single-base homologous regions, and chimeras were removed to achieve accurate impurity removal to ensure the accuracy of the results. After sequencing data that were being preprocessed to generate high-quality sequences, the OTU with sequence similarity ≥97% was defined as a taxon by VSEARCH software (Caporaso et al., 2010). All representative reads were annotated and blasted against the SILVA database (version 132) using RDP classifier v2.11 (confidence threshold of 70%) (Wang et al., 2007). The microbial diversity was estimated using alpha diversity that included the Chao1 index (Chao and Bunge, 2002), Shannon index (Hill et al., 2003), and Simpson index. The UniFrac distance matrix performed by the QIIME software was used for weighted UniFrac principal coordinates analysis (PCoA).

### LC/MS Non-Targeted Metabolomics Analysis

Metabolites were extracted after sample pretreatment. The LC-MS system composed of a Dionex U3000 UHPLC ultra-high performance liquid chromatograph (Thermo Fisher Scientific, USA) and a QE PLUS high-resolution mass spectrometer (Thermo Fisher Scientific, USA) was used as the analytical instrument in this experiment. The operating conditions of the instrument were set as follows: chromatographic conditions—chromatographic column, ACQUITY UPLC HSS T3 (100 mm × 2.1 mm, 1.8 μm); column temperature, 45°C mobile phase, 0.1% formic acid–water (A) and acetonitrile (B); flow rate, 0.35 ml/min; injection volume, 2 μl; and mass spectrum condition—ion source, ESI. The sample mass spectrum signal acquisition adopted the positive and negative ion scanning modes, respectively. After obtaining the original data, Progenesis QI v2.3 software was used to perform standardized preprocessing and qualitative and relative quantitative analyses.

### Statistical Analysis

Statistical analysis was performed using GraphPad Prism software (San Diego, CA, USA), QIIME, and R package (V.2.15.3). Student’s *t*-test and Fisher’s exact test were used to compare sample baseline data. Adonis analysis, Wilcoxon rank-sum test, and Kruskal–Wallis test were used to compare the differences between microbial groups. The metabolomics data were processed and analyzed using the Progenesis QI v2.3 software. Student’s *t*-test and fold change analysis were used to compare metabolites between groups. Pearson correlation coefficient was used to measure the degree of linear correlation between two metabolites. The Spearman rank correlation test was used to assess the correlation between microorganisms and metabolites. *p <*0.05 was considered as statistically significant.

## Results

### Changes in the Structure of the Intestinal Flora Associated With Lung Cancer

This study included 30 lung cancer patients and 15 healthy individuals. The average age of the early-stage lung cancer (ESLC) group, non-early-stage lung cancer (NESLC) group, and healthy control (HC) group was 61.25 ± 6.09, 57.82 ± 9.1, and 59.6 ± 5.45 years old, respectively. No significant difference was found. Similarly, the gender distribution of the three groups has also been verified (Fisher’s exact test, *p* = 0.923). Adenocarcinoma was the main type of lung cancer (6/8 in ESLC, 12/22 in NESLC) (**Table 1**).

**Table 1:** Participants’ baseline data.

Group		ESLC group (*n* = 8)	NESLC group (*n* = 22)	HC group (*n* = 15)	*p*-value
Age, mean ± SD, years		61.25 ± 6.09	57.82 ± 9.1	59.6 ± 5.45	0.519
Gender, male, *n*		4	12	7	0.923
Type				NA	
	Adenocarcinoma	6	12		
	Squamous cell carcinoma	0	6		
	Small cell carcinoma	0	4		
	Large cell carcinoma	1	0		
	Carcinoma *in situ*	1	0		
Tumor staging				NA	
	0	1			
	I	5			
	II	2			
	III		6		
	IV		16		
Tumor metastasis		1	15	NA	

NA, not applicable.

**Figure 1:**
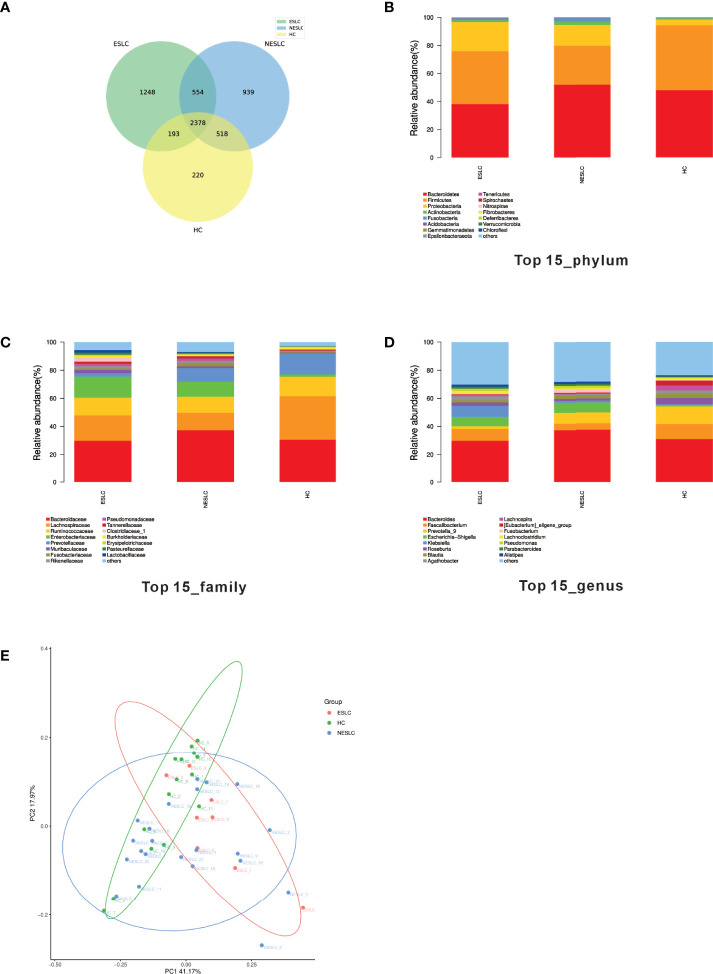
Intestinal flora community structure. **(A)** The Venn diagram showed each group of unique and common OTUs. **(B–D)** The top 15 representative species and their proportions in the three groups at the level of phylum, species, and genus. **(E)** PCoA shows differences between individuals or groups. The abscissa (PC1) and the ordinate (PC2) are the two main coordinates that explain the greatest difference between samples. The points in the graph represent samples, and different colors represent different sample grouping information; similar samples are clustered together.

In order to explore the intestinal microbial composition of lung cancer patients with different stages, we performed 16S rRNA sequencing on stool samples of 30 lung cancer patients (8 of ESLC, 22 of NSLC) and 15 healthy individuals. The VSEARCH (version 2.4.2) software (Rognes et al., 2016) was used to classify OTU according to 97% of similarity. The sequence with the largest abundance in each OTU was selected as the representative sequence of the OTU, compared, and annotated with the SILVA (v132) database using the RDP classifier (v2.11) as the annotation tool. A total of 6,053 annotated OTUs were obtained. Compared with HC, the total OTUs and unique OTUs of ESLC and NESLC increased, and there were 2,378 annotation OTUs shared by the three groups (**Figure 1A**).

We analyzed the community structure of intestinal microbial (**Supplemental Material 1**). At the phylum level, *Bacteroides*, *Firmicutes*, and *Proteobacteria* were the main components in the three groups. At the family level, besides similar families with higher abundance (*Bacteroidaceae*, *Lachnospiraceae*, *Ruminococcaceae*), the abundance of *Enterobacteriaceae* was higher in ESLC (15.01%), *Enterobacteriaceae* and *Prevotellaceae* in NESLC (10.55%, 9.71%), and *Prevotellaceae* in HC (14.87%). At the genus level, apart from the similar higher abundance genus (*Bacteroides*), *Faecalibacterium* (10.71%), *Prevotella_9* (12.56%), and *Bacteroides* (7.33%) were the most abundant genera identified in HC; *Faecalibacterium* (8.25%), *Klebsiella* (7.76%), and *Escherichia–Shigella* (6.77%) in ESLC; and *Prevotella_9* (7.77%) and *Escherichia–Shigella* (7.23%) in NESLC (**Figures 1B–D**).

The microbial abundance was statistically analyzed (**Supplemental Material 2**). The Chao1 index showed that there was no significant difference in community richness among groups, while the Shannon and Simpson indexes both showed that each group had similar community diversity. When comparing the structure of the microbial community, β diversity showed differences among the groups (**Figure 1E**, Adonis, *p* = 0.021).

### Analysis of Differences in Intestinal Microbes

The Kruskal–Wallis algorithm was used to further identify microbes with different abundances, and 3 phyla, 21 families, and 55 genera were identified (**Supplemental Material 3**). At the genus level, *Escherichia–Shigella*, *Anaerotruncus*, *Ruminiclostridium*, *Lactobacillus*, *Pediococcus*, *Sphingobium*, *Prevotellaceae*, *Prevotella_1*, and *Olsenella* were rich in the two lung cancer groups and also *Cryptobacterium* in ESLC and *Lachnospira*, *Roseburia*, *Brevundimonas*, *Lachnospiraceae_UCG-006*, and *Lachnospiraceae_UCG-004* in HC.

**Figure 2:**
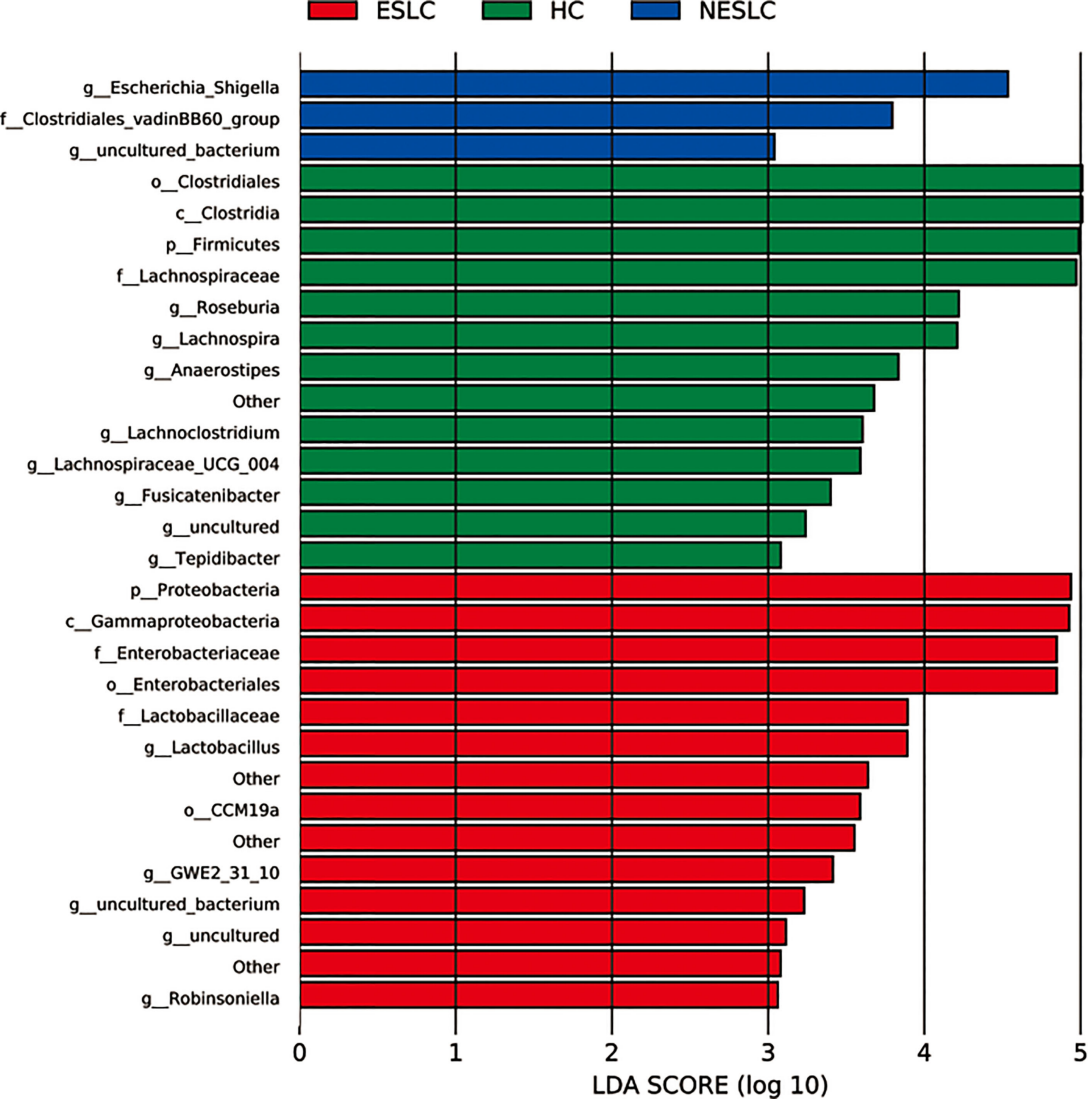
Key genera selection. Differential microbial score chart: the higher the score, the greater the contribution of the microbe to the difference.

Linear discriminant analysis (LDA) effect size (LEfSe) was used to identify key microbial taxa. Eight, five, and two key genera were identified in HC, ESLC, and NESLC, respectively (**Figure 2**). The genus with an average relative abundance of less than 0.01% was excluded. The key species were *Roseburia* (LDA score 4.22, *p* = 0.012), *Lachnospira* (LDA score 4.21, *p* = 0.001), *Anaerostipes* (LDA score 3.83, *p* = 0.007), and *Lachnoclostridium* (LDA score 3.60, *p* = 0.042) in HC; *Lactobacillus* in ESLC (LDA score 3.89, *p* = 0.029); and *Escherichia_Shigella* in NESLC (LDA score 4.53, *p* = 0.010).

### Changes of Plasma Metabolite Profile in Lung Cancer Patients and Crucial Metabolites

**Figure 3:**
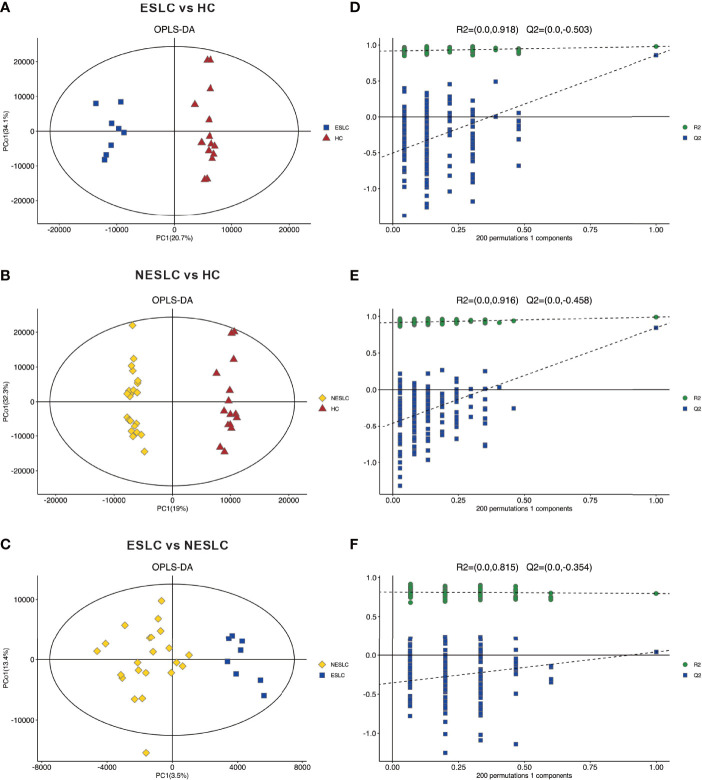
Principal component analysis. **(A–C)** OPLS-DA score chart shows the difference in metabolites between groups. The abscissa represents the variation between groups, and the ordinate represents the variation within groups. **(D–F)** Comparison of the true model parameters in the validation test and those of permutated models.

Metabolites and fermentation products produced by intestinal flora can enter the blood and have a functional impact on the host’s physiology. Therefore, in order to further explore the changes in the intestinal microbe–host interaction, we examined the metabolic profile in the serum. Based on the abundance of metabolites detected by non-targeted metabolomics, the orthogonal partial least-squares discriminant analysis (OPLS-DA) was performed. According to the scatter plot, samples from different groups were largely separable, indicating different metabolic patterns (**Figures 3A**–**C**). The permutation test showed that there was no overfitting to the data, and verified the OPLS-DA model (**Figures 3D**–**F**). Generally, the closer the slopes of the R2Y and Q2Y lines were to zero, the more likely the model was to be overfitted. A total of 5,514 metabolites were identified, consisting of 2,793 of positive ion and 2,721 of negative ion (**Supplemental Material 4**).

**Figure 4:**
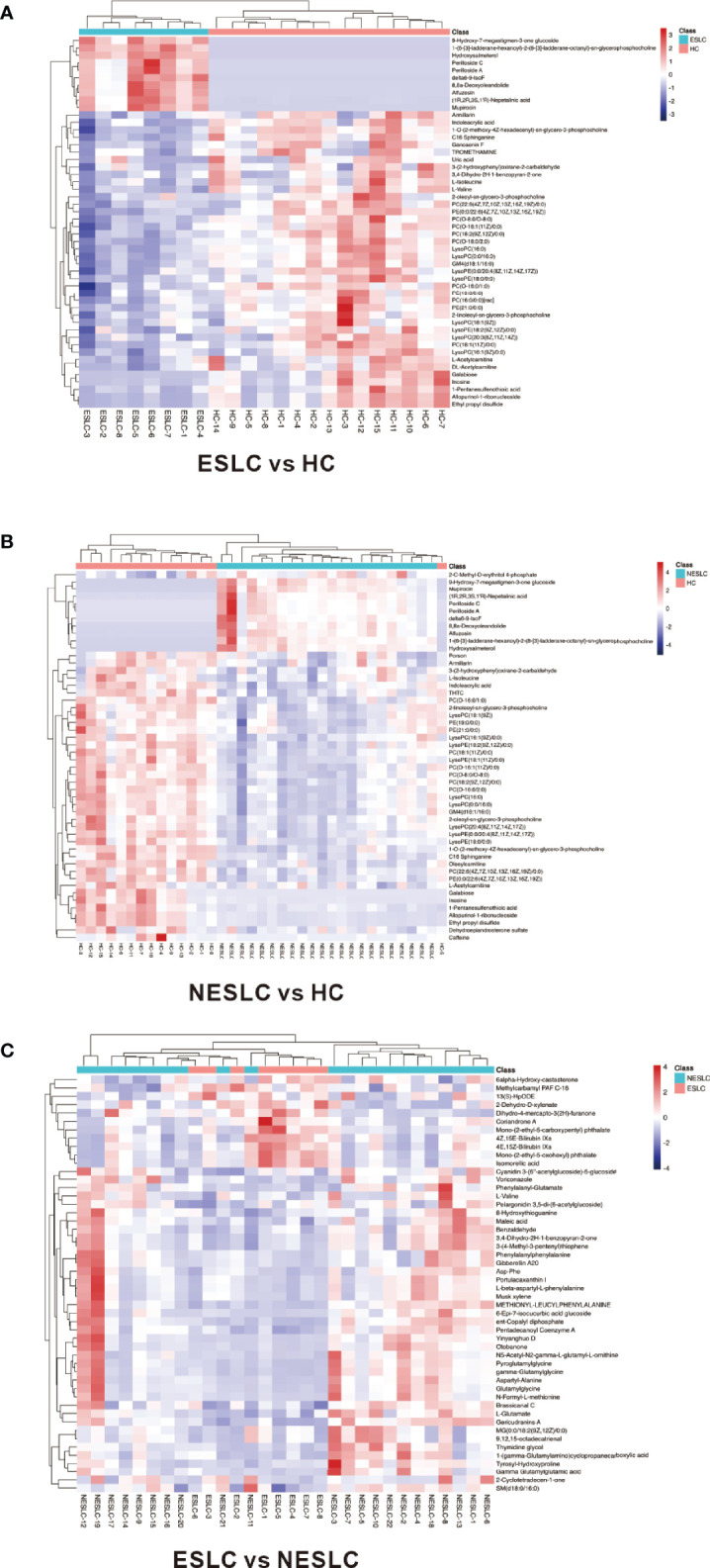
Heatmap of differential metabolites. **(A)** Differential metabolites in ESLC vs. HC. **(B)** Differential metabolites in NESLC vs. HC. **(C)** Differential metabolites in ESLC vs. NESLC. The abscissa represents the sample name, and the ordinate represents the differential metabolite. The color from blue to red indicates that the expression abundance of metabolites is from low to high.

Variable importance of projection (VIP) value was obtained through the OPLS-DA model. The biologically significant differential metabolites were mined according to the screening criteria: the VIP value of the first principal component of the OPLS-DA model >1 and the *p-*value of the *t*-test <0.05. The larger the value, the greater the contribution of the variable to the grouping. ESLC vs. HC, NESLC vs. HC, and ESLC vs. NESLC screened out 272, 319, and 68 differential metabolites, respectively (**Supplemental Material 5**). Hierarchical clustering was performed on the expression of the differential metabolites with the top 50 of VIP value to show the relationship among samples and the expression differences of metabolites among samples more intuitively. The result is shown below (**Figure 4**). 9-Hydroxy-7-megastigmen-3-one glucoside, 1-[6-(3)-ladderane-hexanoyl-2-(8-(3)-ladderane-octany])-sn-glycerophosphocholine, and perilloside A were more abundant in the lung cancer groups, while indoleacrylic acid, L-isoleucine, L-valine, PC(O−16:0/2:0), and LysoPC (16:0) decreased.

Correlation analysis can help measure the correlation between differential metabolites. Differential metabolites with the top 50 of VIP value were selected for visual analysis. It was found that perilloside C, which was richer in the lung cancer groups, was negatively correlated with PC(O−16:0/2:0), which was richer in the HC group (**Supplemental Material 6**).

**Figure 5:**
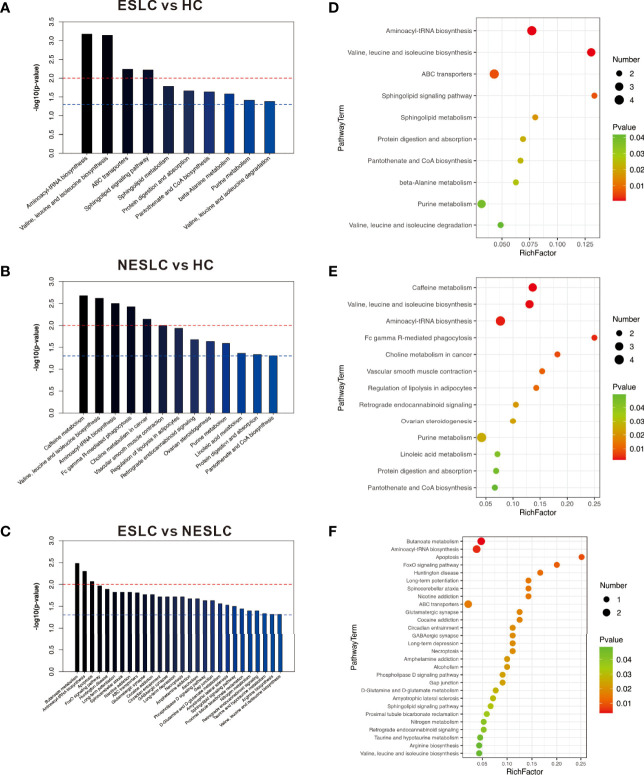
**(A–C)** The *p*-value is the significance of enrichment in metabolic pathways. The red line indicates that the *p*-value is 0.01, and the blue line indicates that the *p*-value is 0.05. When the top of the bar is higher than the blue line, the signal pathway it represents is significant. **(D–F)** The ordinate is the name of the metabolic pathway, and the abscissa is the rich factor (rich factor = the number of significantly different metabolites/the total number of metabolites in the pathway); the greater the rich factor, the greater the degree of enrichment. The color from green to red indicates that the *p*-value decreases sequentially; the larger the point, the more metabolites enriched on the pathway.

The KEGG ID of the metabolites was used for pathway enrichment analysis. The differential metabolites of ESLC vs. HC were mainly involved in aminoacyl-tRNA biosynthesis; valine, leucine, and isoleucine biosynthesis; ABC transporters; and sphingolipid signaling pathway (*p* < 0.01); those of NESLC vs. HC were involved in caffeine metabolism; valine, leucine, and isoleucine biosynthesis; aminoacyl-tRNA biosynthesis; Fc gamma R-mediated phagocytosis; and choline metabolism in cancer (*p* < 0.01). On the other hand, the differential metabolites of ESLC vs. NESLC were involved in butanoate metabolism, aminoacyl-tRNA biosynthesis, and apoptosis (*p* < 0.01) (**Figure 5**).

**Figure 6:**
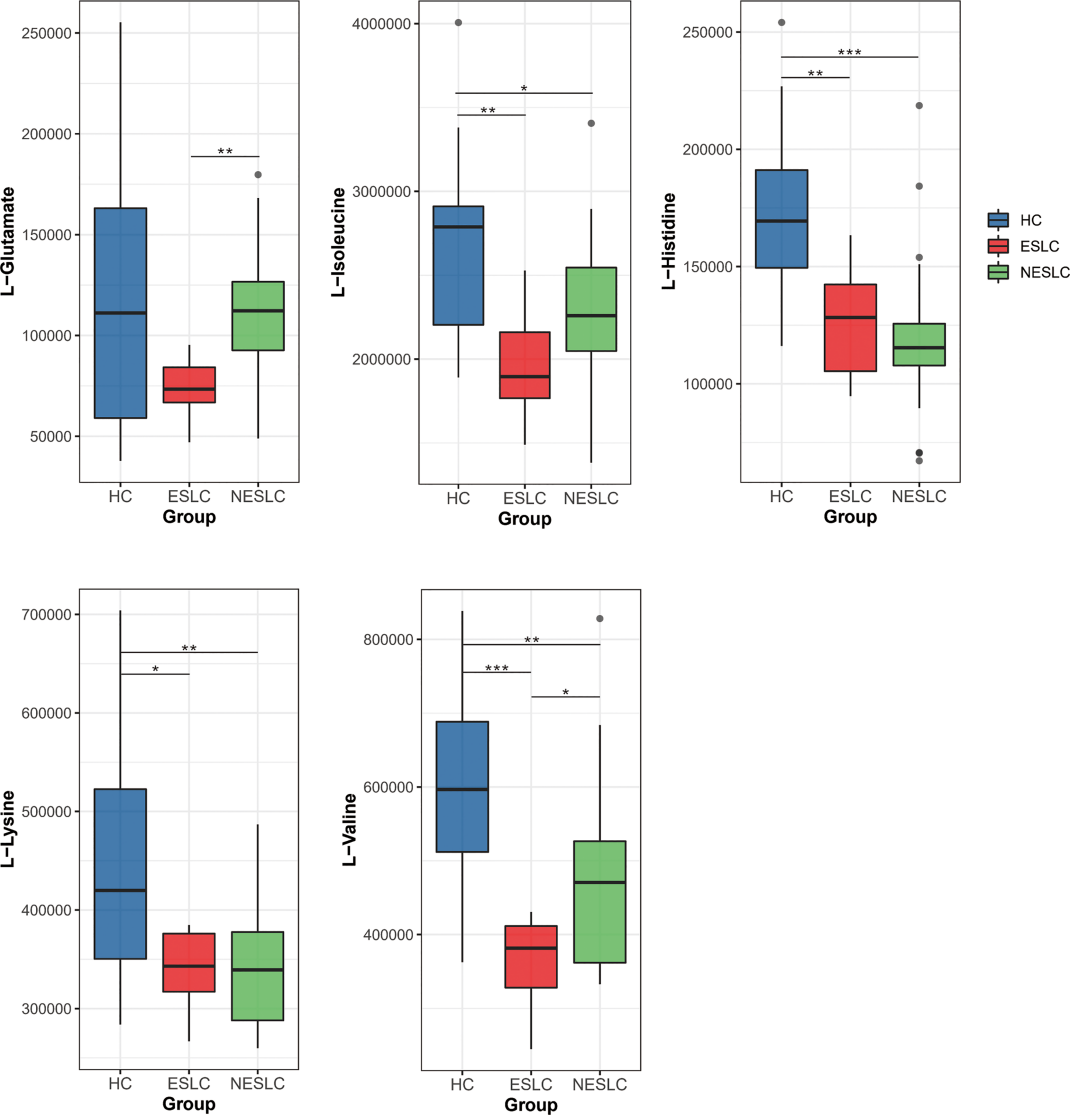
Distribution of different metabolites in each group. *p < 0.05, **p < 0.01, ***p < 0.001.

We found that the aminoacyl-tRNA biosynthesis pathway seemed to be closely related to the progression of lung cancer, so we focused on the differential metabolites involved in it, L-valine, L-lysine, L-isoleucine, L-histidine, and L-glutamate, to seek new biomarkers (**Supplemental Material 7**). We reviewed the expression levels of these metabolites in different samples (**Figure 6**). The results showed that L-lysine, L-isoleucine, and L-histidine decreased significantly in the lung cancer groups. Compared with ESLC, L-glutamate increased significantly in NESLC. L-valine showed significant differences in the pairwise comparisons, and the trend of change was first down and then up, but always lower than the healthy level.

### Conjoint Analysis

**Figure 7:**
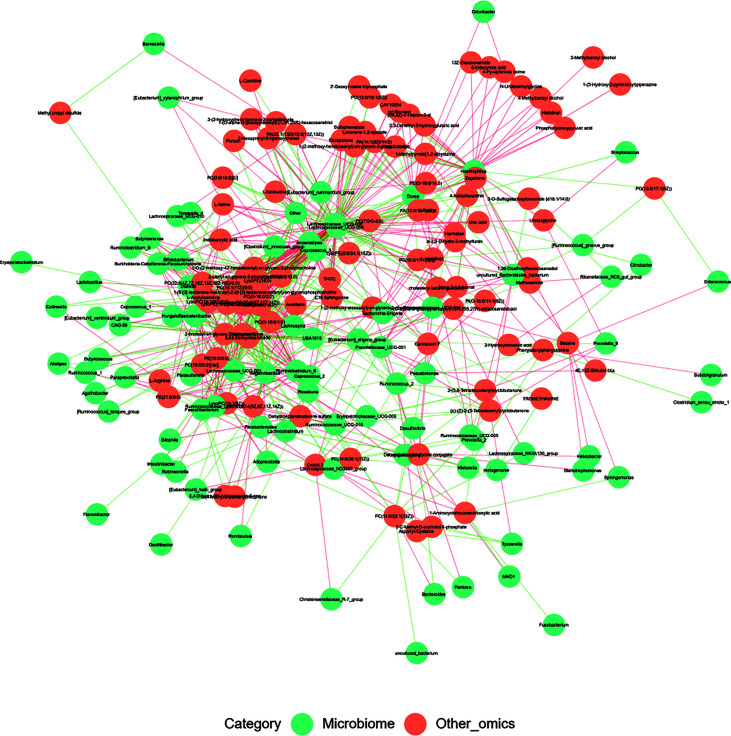
The CorNetwork diagram. The connection indicates correlation. The red connecting line represents a positive correlation between nodes, while the blue line represents a negative correlation.

Correlation analysis was performed to help us better understand the correlation between the microbiome and plasma metabolome. Based on metabolomics and genomics datasets, network analysis was performed to determine broader associations between the two (**Figure 7** and **Supplemental Material 8**). The CorNetwork diagram showed the relationship between *Anaerostipes*, *Coprococcus_3*, *Lachnospiraceae_UCG-004*, *Lachnospiraceae_UCG-006*, and various metabolites. The Spearman correlation coefficients between the top 100 differential metabolites and the top 100 differential microbes were calculated (**Supplemental Material 9**). The results showed that there was a significant correlation between L-valine and *Lachnospira*, *Anaerostipes*, *Coprococcus_3*, *Fusicatenibacter*, *Lachnospiraceae_UCG-004*, *[Eubacterium]_xylanophilum_group*, *Lachnospiraceae_UCG-006*, and *Burkholderia–Caballeronia–Paraburkholderia*. Among them, *Lachnospiraceae_UCG-006* was the most correlated genus (correlation = 0.49, *p* < 0.001), which was also a differential genus enriched in HC.

## Discussion

Our research has proved the changes of intestinal microbiota and serum metabolic spectrum in lung cancer patients. We combined the two omics to find the possible pathogenesis and potential biomarkers of lung cancer.

The control group consisted of individuals undergoing physical examination from the Health Management Center, while lung cancer patients were newly diagnosed without treatment. Medication use is an important consideration when we screen study subjects, which is mainly because any medication, such as antibiotics and anticancer medication, will cause changes in intestinal flora to varying degrees. We have observed differences in the composition and structure of gut microbes between lung cancer patients with different stages and healthy people. *Faecalibacterium* has a higher abundance in HC, and its relative and absolute abundances in ESLC and NESLC decreased sequentially. *Faecalibacterium* is considered to be a marker of healthy intestines and is a type of butyrate-producing bacteria (Wang et al., 2007). In previous studies, it was also found that its abundance decreased in lung cancer patients (Gui et al., 2020; Zheng et al., 2020). In addition, ESLC has abundant *Klebsiella* and *Escherichia–Shigella*, while NESLC has abundant *Prevotella_9* and *Escherichia–Shigella*. *Klebsiella*, *Escherichia–Shigella*, and *Prevotella_9* all contain many opportunistic pathogens. The latest research has linked the increase in *Prevotella* abundance with local and systemic diseases, including rheumatoid arthritis, hypertension, and metabolic disorders (Horta-Baas et al., 2017; Li et al., 2017; Ding et al., 2020).

Previous studies have shown that the changes of intestinal flora in patients with lung cancer are often related to *Firmicutes*, *Bacteroides*, and *Proteobacteria* (Zhang et al., 2018; Liu et al., 2019; Zhuang et al., 2019; Zheng et al., 2020; Georgiou et al., 2021). When using the Kruskal–Wallis algorithm to further identify microbes with different abundances, it was found that the abundance of *Firmicutes* decreased and *Proteobacteria* increased in the lung cancer group. Compared with previous studies, the change of *Firmicutes* was consistent and *Proteobacteria* was also consistent with most studies, which only decreased in the study of Zhang et al. However, *Bacteroides* has been found to increase in lung cancer, while *Actinomycetes* has been found to decrease (Zhuang et al., 2019; Zheng et al., 2020), which was not found in our study, even in the comparison between HC and NESLC. We try to find the reasons for the differences from the included population and experimental design. The study of Zheng et al. included patients with early-stage lung cancer. Although one of its exclusion criteria was antibiotic use in the last 8 weeks, the use of anticancer drugs was not specified. In the study of Zhuang et al., subjects did not use any drugs in the past 3 months and seemed to have not received chemotherapy, but it was uncertain whether there were therapeutic behaviors other than chemotherapy. Our subjects were all newly diagnosed and treated patients, so we consider that the difference may be due to the selection criteria of subjects or the heterogeneity of patients. It is worth mentioning that although no difference was found at the level of *Bacteroides* and *Actinomycota*, differential genera mined such as *Roseburia*, *Lachnospira*, *Olsenella*, and *Cryptobacterium* belonged to *Bacteroides or Actinomycota*.

When looking for key differential microorganisms, it was found that *Anaerostipes*, *Lachnoclostridium*, *Roseburia*, and *Lachnospira* were the key differential genera in HC. These genera are closely related to the production of short-chain fatty acids (Liu et al., 2019; Chen et al., 2021; Yi et al., 2021). Short-chain fatty acids play an important role in human health because they regulate the function and differentiation of almost all intestinal immune cells (Surono et al., 2021). It has been found that short-chain fatty acids maintain intestinal homeostasis by promoting the production of IL-10 in Th1 cells (Sun et al., 2018). Xia et al. (2020) made preparations from plant flower buds to induce SCFA-producing bacteria to produce SCFAs to achieve an anticancer effect. Correspondingly, the key differential genus in ESLC was *Lactobacillus*. *Lactobacillus* itself is a kind of beneficial bacteria, which acts in preventing pathogenic bacteria from invading and colonizing the intestines, enhances the body’s immunity, and has anticancer effects (Hendler and Zhang, 2018; Eslami et al., 2019; Han et al., 2021). In colorectal cancer, *Lactobacillus* showed reduced abundance (Loftus et al., 2021). However, in a large cohort study, it was found that the increased abundance of *Lactobacillus* bacteria in the oral cavity was closely related to the risk of lung cancer (Hosgood et al., 2021). The key differential genus in NESLC was *Escherichia_Shigella*. The increased abundance of *Escherichia/Shigella* was considered to be a characteristic of intestinal flora imbalance in Crohn’s disease (Pascal et al., 2017). These indicate that compared with HC, patients with early/advanced lung cancer have different degrees of intestinal flora imbalance. Random forest algorithm, a kind of machine learning algorithm, was used to screen important microorganisms that distinguish differences between groups. The screened genera were mostly the same as the key genera in each group. However, it should be noted that due to the hierarchical structure of the algorithm, there may be no linear relationship between the filtered features and the output. For example, *Pseudomonas*, although selected as the most important genus, was not a differential genus, and the difference between groups was not statistically significant.

Pathway enrichment analysis was performed using differential metabolites to understand the mechanism of metabolic pathway changes in different samples. Results showed that the aminoacyl-tRNA biosynthesis pathway was enriched in HC vs. ESLC, HC vs. NESLC, and ESLC vs. ESLC, which seemed to be closely related to the progression of lung cancer. In previous studies, the aminoacyl-tRNA biosynthesis pathway was an enrichment pathway for UAP1 expression-related genes in lung adenocarcinoma (Wang et al., 2020). It was closely related to cisplatin resistance in non-small cell lung cancer (Shi et al., 2019). Moreover, it has also been found to be upregulated in gastric cancer, and researchers even proposed a new therapeutic strategy for gastric cancer targeting the aminoacyl-tRNA biosynthesis pathway (Gao et al., 2021). Differential metabolites in this pathway, L-lysine, L-isoleucine, and L-histidine, were significantly reduced in the lung cancer groups, but there was no significant difference between ESLC and NESLC, which meant that these indicators could not be used as reference indicators for staging. L-glutamate was only significantly different between ESLC and NESLC; thus, it was not the best predictor. The serum concentration of L-valine showed differences in the pairwise comparison, so it may be used as a potential marker. In the correlation analysis, we found that the most strongly related genus of L-valine was *Lachnospiraceae_UCG-006*, which was the differential genus we screened earlier. The level of valine in early-stage non-small cell lung cancer was lower than that in advanced stage (Puchades-Carrasco et al., 2016). In the study of Ni et al. (2019), valine declined in the lung cancer group as a potential biomarker, although this research was based on two existing data sets. In addition, valine has been reported as a potential biomarker for the differential diagnosis of seronegative rheumatoid arthritis and psoriatic arthritis (Souto-Carneiro et al., 2020). Upregulated concentrations of branched-chain amino acids were detected in stool samples from colorectal cancer patients and in gastric tissue fluid from mice with gastric cancer (Yachida et al., 2019; Gu et al., 2020).

It is worth mentioning that although this study describes the changes in the microbiome and metabolome in lung cancer and the relationship between the two, we cannot explain the causal relationship between them. This requires more and larger cohort studies to explore in the future.

This study has some limitations. Prognosis is an important part of disease research. However, due to the inability to track the prognosis of all patients in the short term, we are temporarily unable to study the prognosis of the disease. On the other hand, the strict entry conditions lead to a small number of participants. We hope to expand the sample size and conduct further studies through multicenter cooperation in our future research.

## Conclusion

This study describes the characteristics of intestinal flora and serum metabolic profiles of patients with lung cancer in different stages. It reveals that lung cancer may be the result of the mutual regulation of L-valine and *Lachnospiraceae_UCG-006* through the aminoacyl-tRNA biosynthesis pathway, and proposes that L-valine may be a potential marker for the diagnosis of lung cancer.

## Data Availability Statement

The datasets presented in this study can be found in online repositories. The names of the repository/repositories and accession number(s) can be found below: https://data.mendeley.com/datasets/8rftx9ybnm/1, genomic sequencing; https://data.mendeley.com/datasets/nj4cz7mmj5/1, metabolomics.

## Ethics Statement

The studies involving human participants were reviewed and approved by the IRB of The Third Xiangya Hospital of Central South University. The patients/participants provided their written informed consent to participate in this study.

## Author Contributions

Design of the study: SC, RG, and Y-FF. Methodology: SC and X-HZ. Formal analysis: SC and X-HZ. Data curation: SC and X-HZ. Software: SC. Writing—original draft preparation: SC and X-HZ. Writing—review and editing: J-HZ, H-YJ, H-TL, SC, RG, and Y-FF. All authors contributed to the article and approved the submitted version.

## Funding

This work was supported by the National Natural Science Foundation of China (Grant No. 82002452), and the Fundamental Research Funds for the Central Universities of Central South University (Grant No. 2021zzts1090).

## Conflict of Interest

The authors declare that the research was conducted in the absence of any commercial or financial relationships that could be construed as a potential conflict of interest.

## Publisher’s Note

All claims expressed in this article are solely those of the authors and do not necessarily represent those of their affiliated organizations, or those of the publisher, the editors and the reviewers. Any product that may be evaluated in this article, or claim that may be made by its manufacturer, is not guaranteed or endorsed by the publisher.
