# Supplementary figures and images for: Combined Microbiome and Metabolome Analysis Reveals a Novel Interplay Between Intestinal Flora and Serum Metabolites in Lung Cancer
Source: Front Cell Infect Microbiol. 2022 May 2;12:885093. doi: 10.3389/fcimb.2022.885093 (PMC9108287; doi:10.3389/fcimb.2022.885093)

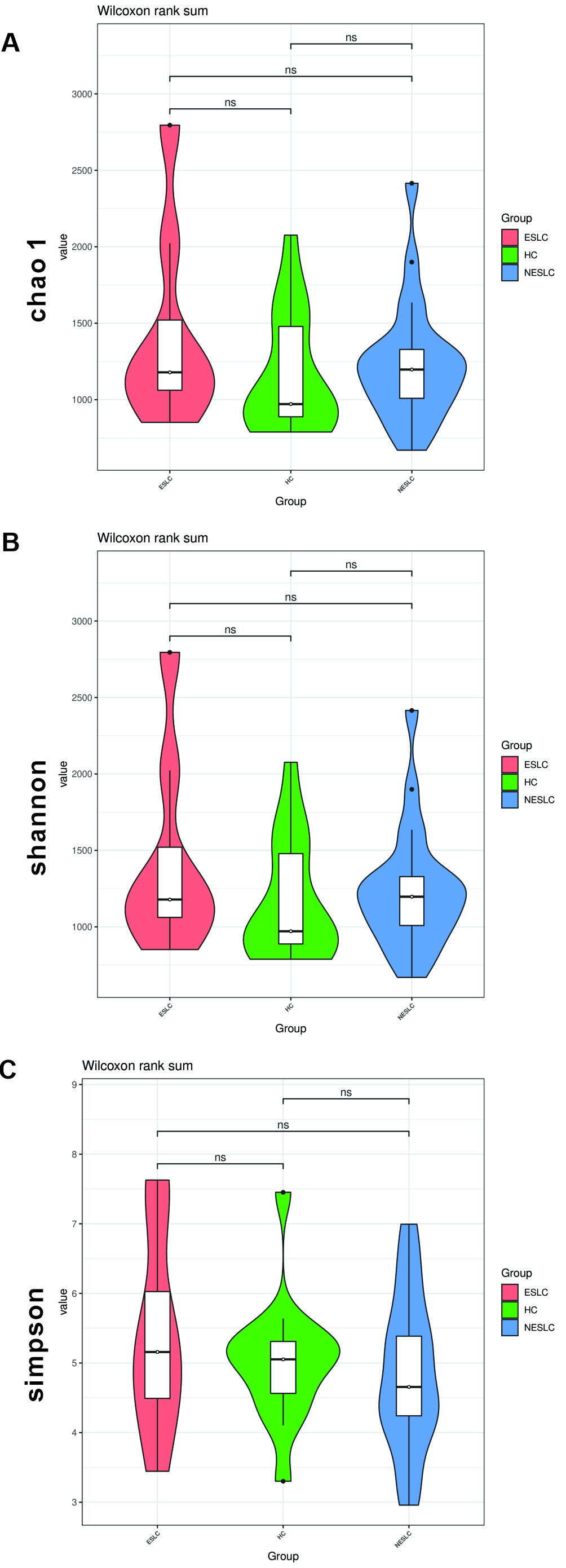

Supplement: Supplementary file 1 [file DataSheet_1.zip › Supplemental Material 2.tif]

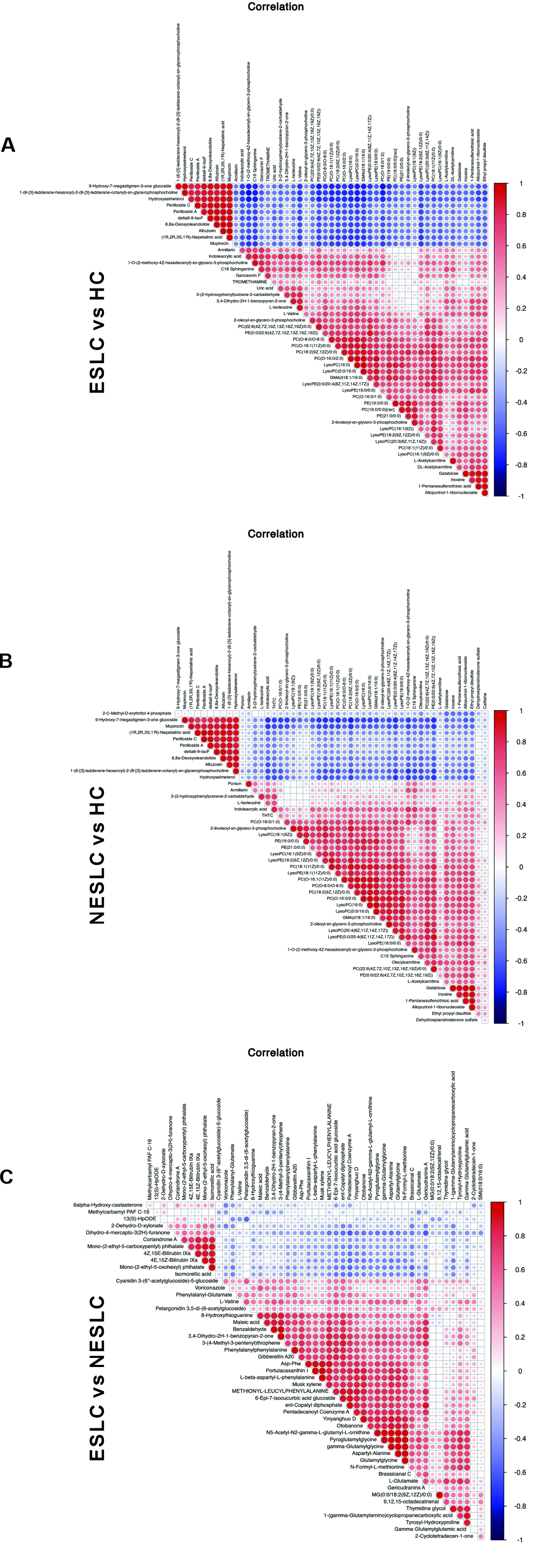

Supplement: Supplementary file 1 [file DataSheet_1.zip › Supplemental Material 6.tif]

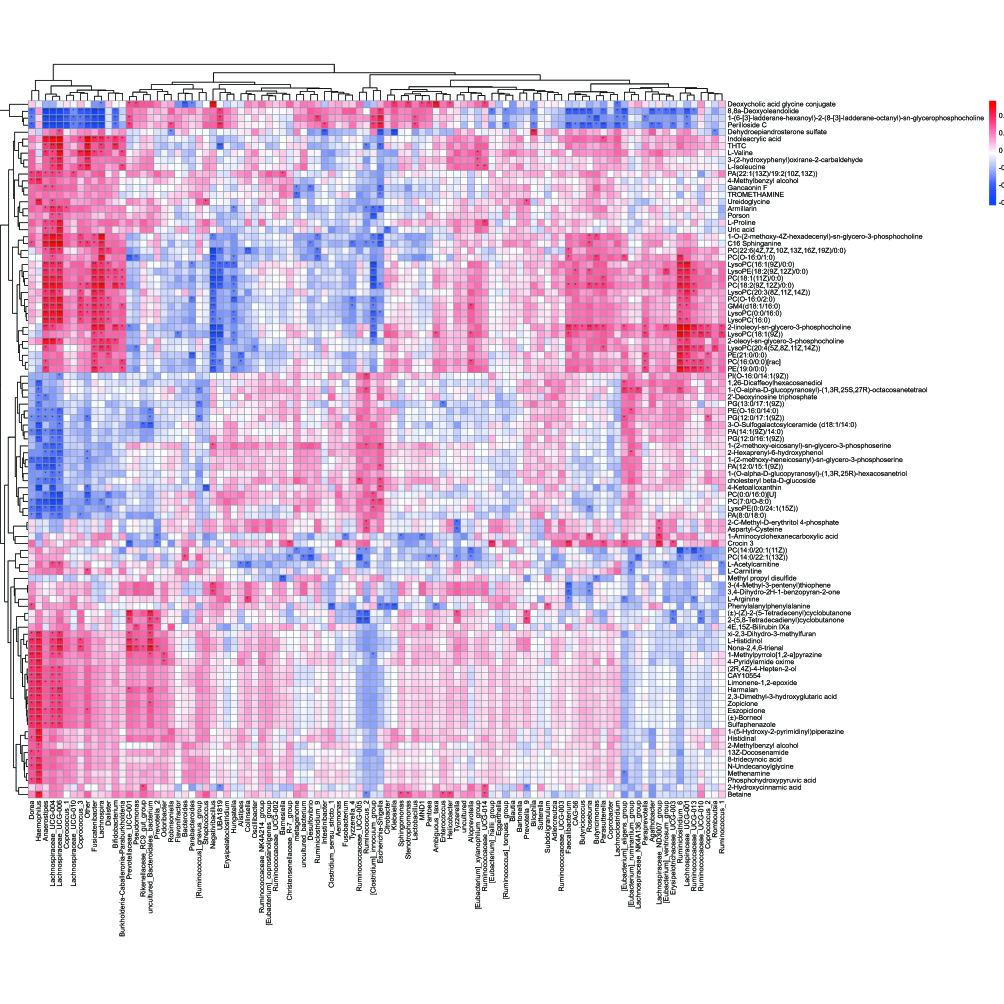

Supplement: Supplementary file 1 [file DataSheet_1.zip › Supplemental Material 9.tif]
